# An Obesogen over Time: Transgenerational Impact of Tributyltin

**DOI:** 10.1289/ehp.121-a96

**Published:** 2013-03-01

**Authors:** Wendee Nicole

**Affiliations:** Wendee Nicole, based in Houston, TX, has written for *Nature*, *Scientific American*, *National Wildlife*, and other magazines.

The term “obesogen” describes a chemical that promotes excessive weight gain by increasing adipocyte (fat cell) size or number, changing metabolism to favor fat storage, or altering the control of appetite and satiety. Previous studies have suggested that prenatal exposure to obesogens can alter metabolism in mice and predispose multipotent mesenchymal stem cells (MSCs) to become fat cells, but whether these changes last through subsequent generations was unknown. A team of researchers investigating this question exposed pregnant mice to tributyltin (TBT) and found impacts on adipogenesis through the third generation [*EHP 121(3):359–366; Chamorro-Garcia et al.*].

TBT, which has been used as a biocide in marine paints and in textile products such as carpets, is a persistent organic pollutant that is ubiquitous in the environment. Exposure to TBT is thought to occur through many sources, including food, house dust, and consumer products.

In the current study, male offspring (i.e., the F1 generation) of mice exposed to TBT during pregnancy showed marked increases in the number and size of white fat cells and significant but less pronounced increases in the weight of white fat deposits (or “depots”) around the kidney and under the skin. The two subsequent generations (F2 and F3) also showed increases in fat cell size and number and, at the two higher TBT doses, in fat depot weight. Females showed more modest differences following TBT prenatal exposure, including both increases and decreases in cell numbers, depending on location.

**Figure:**
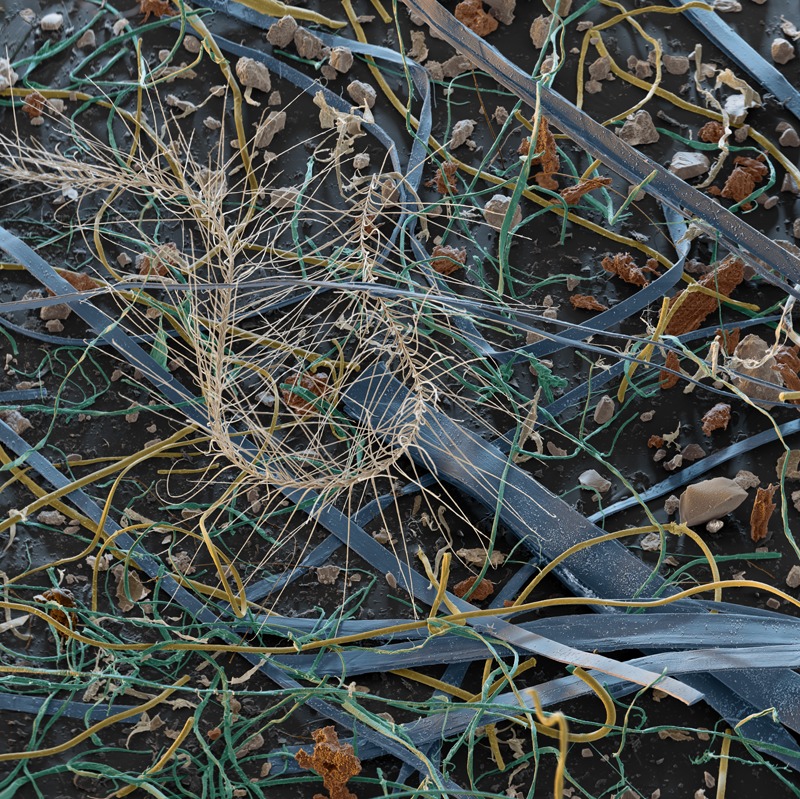
Once used to fight mites and fungi in carpets and other textiles, TBT has been found in house dust, which is thought to be a major source of human exposure. This dust sample—which was not tested for TBT—is made up of carpet fibers (blue), cotton fibers (green), synthetic fibers (yellow), small stones (gray), dirt particles (dark brown), skin flakes (beige), and a feather. © Eye of Science/Science Source

The team also found that F3 animals in the TBT group showed substantially increased expression of adipogenic markers in bone marrow–derived MSCs and decreased expression of osteogenic (bone lineage) markers—in other words, the stem cell population appeared reprogrammed to create fat instead of bone. The shift was more pronounced in males in all generations, and especially in the F3 generation.

When the team looked at accumulation of lipids in the livers of each generation, they found that TBT-exposed F1 males and females showed marked increases in lipid accumulation; F2 and F3 generations also showed less pronounced increases in liver lipids. The investigators also found significant increases in the expression of hepatic genes related to fat creation and storage in males and females from all three generations of mice.

F1 animals were exposed to TBT as fetuses, and F2 animals were potentially exposed as germ cells within the F1 fetuses. F3 animals were not exposed to TBT, yet they exhibited signs characteristic of exposure. This is the first known study to show that prenatal exposure to low, environmentally relevant concentrations of an obesogen led to transgenerational effects on white fat volume, altered stem cell programming, and liver fat accumulation in multiple generations of mice.

